# Persistence of viral RNA in North American elk experimentally infected with an ancestral strain of severe acute respiratory syndrome coronavirus 2 (SARS-CoV-2)

**DOI:** 10.1038/s41598-024-61414-7

**Published:** 2024-05-15

**Authors:** Paola M. Boggiatto, Alexandra Buckley, Eric D. Cassmann, Hannah Seger, Steven C. Olsen, Mitchell V. Palmer

**Affiliations:** 1 Infectious Bacterial Diseases Research Unit, National Animal Disease Center, USDA, Agricultural Research Service, Ames, IA USA; 2 Virus and Prion Research Unit, National Animal Disease Center, USDA, Agricultural Research, Ames, IA USA; 3 Oak Ridge Institute for Science and Education, 1299 Bethel Valley Rd., Oak Ridge, TN 37830 USA

**Keywords:** Viral pathogenesis, Pathogens, Humoral immunity, Immunology, Infection

## Abstract

White-tailed deer (*Odocoileus virginianus*) have emerged as a reservoir host for SARS-CoV-2 given their susceptibility to infection and demonstrated high rates of seroprevalence and infection across the United States. As SARS-CoV-2 circulates within free-ranging white-tailed deer populations, there is the risk of transmission to other wildlife species and even back to the human population. The goal of this study was to determine the susceptibility, shedding, and immune response of North American elk (*Cervus elaphus canadensis*) to experimental infection with SARS-CoV-2, to determine if another wide-ranging cervid species could potentially serve as a reservoir host for the virus. Here we demonstrate that while North American elk do not develop clinical signs of disease, they do develop a neutralizing antibody response to infection, suggesting the virus is capable of replicating in this mammalian host. Additionally, we demonstrate SARS-CoV-2 RNA presence in the medial retropharyngeal lymph nodes of infected elk three weeks after experimental infection. Consistent with previous observations in humans, these data may highlight a mechanism of viral persistence for SARS-CoV-2 in elk.

## Introduction

Previous work from our laboratory identified white-tailed deer (*Odocoileus virginianus*) as a susceptible host for severe acute respiratory syndrome coronavirus 2 (SARS-CoV-2), capable of transmission^1,2^. Subsequent field work demonstrated a high level of seroprevalence and infection in free-ranging white-tailed deer across the United States (US)^3–8^. Evidence of multiple^4,8^ as well as new^9^ variants within white-tailed deer populations has been presented. Additionally, intra- as well as inter-species transmission (i.e. spillback into humans)^5,10^ has shown that white-tailed deer could serve as an important reservoir host for SARS-CoV-2 evolution and transmission.

Interest in determining whether white-tailed deer were susceptible to SARS-CoV-2 infection evolved from a comparative analysis of vertebrate angiotensin-converting enzyme 2 (ACE2) receptor genes, the main receptor for SARS-CoV-2. Based on these analyses, the white-tailed deer ACE2 receptor sequence showed a high degree of homology with the human ACE2 receptor and was classified as having a high propensity for binding the Spike protein of the virus^11,12^. Two additional cervid species, reindeer (*Rangifer tarandus*) and Pere David’s deer (*Elaphurus davidandus*) were also predicted to express ACE2 receptors with a high propensity for binding. North American elk (*Cervus elaphus canadensis*), another cervid species, was not reported as a possible susceptible host based on ACE2 receptor homology. Field surveillance of SARS-CoV-2 seroprevalance in red deer (*Cervus elaphus*), a closely related species to North American elk, found no evidence of exposure in Europe^13–15^. Data from these studies also suggested that other free-ranging deer species in Europe including Fallow (*Dama dama*), Muntjac (*Muntiacus reevesi*), Sika (*Cervus nippon*) and Roe deer (*Capreolus capreolus*) were also seronegative^13^.

The high seroprevalence observed in white-tailed deer populations can be attributed to both susceptibility to infection but also to sources of infection from humans. As SARS-CoV-2 circulates in white-tailed deer, they may serve as a source of infection for other species. In the US, elk are primarily found in the western part of the US, with successful reintroductions in 8 states east of the Mississippi river including Wisconsin, Michigan, Minnesota, Missouri, Pennsylvania, Arkansas, Kentucky, Tennessee, West Virginia, Virginia, and North Carolina. The overall population in North America is approximated at one million elk^16^. Elk are gregarious animals that tend to spend most of the time in herds, except for adult bulls that lead solitary lives outside of the rut season. In the West, artificial feeding grounds during winter months cause large aggregations of elk, which can affect infectious disease spread with the herd (reviewed in^17^). Furthermore, elk can serve as hosts for a variety of viral, bacterial and prion diseases, and the risk for spillover into domestic livestock is well documented^18^. Similarly, although not as common, cases of disease outbreaks in humans have been reported that are directly tied to elk^19,20^. While elk may not currently have the same wide distribution of white-tailed deer, human-elk interactions do exist including through recreational practices such tourism, hunting, and animal watching, and through encroachment and co-existence on elk habitat, including migration routes. Therefore, we sought to determine the susceptibility of North American elk to SARS-CoV-2 infection.

Recently, a study by Porter et al.^21^ demonstrated that weanling elk are minimally susceptible to infection with the Delta variant of SARS-CoV-2. While they did not develop clinical signs and were not capable of onward transmission, the elk did develop a low-level antibody response as measured by plaque reduction neutralization test. In the work presented here, we expand on this initial study in North American elk and assess the susceptibility of elk calves and adults to the ancestral Wuhan-like strain of SARS-CoV-2 (USA-WA1/2020). We provide further evidence that while North American elk do not develop clinical signs associated with infection, SARS-CoV-2 does elicit a neutralizing antibody response, suggesting that the virus is capable of establishing infection in this species. Additionally, we show that SARS-CoV-2 RNA persists in the lymph node of infected animals in the absence of viral protein. These findings provide additional information regarding host–pathogen interaction mechanisms for SARS-CoV-2 in one of its many hosts.

## Results

### Development of neutralizing antibody responses against SARS-CoV-2

**Figure 1:**
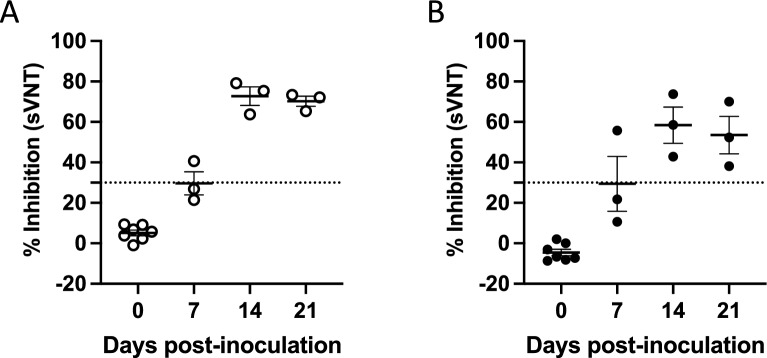
Presence of SARS-CoV-2 neutralizing antibodies in serum from calves and adult elk. Serum samples were collected at various timepoints following intranasal infection with SARS-CoV-2 and assessed via sVNT for antibodies against the virus. Shown are percent inhibition results for (**A**) elk calves and (**B**) adult elk. Bars indicate mean percent inhibition values, and error bars indicate ± SD. Dotted line indicates assay cut off for positive results.

Blood samples were collected on days 0, 7, 14 and 21 post-inoculation (p.i.) to assess neutralizing antibodies against SARS-CoV-2. In both calves (Fig. 1A) and adult (Fig. 1B) elk, antibody titers to SARS-CoV-2 measured by surrogate virus neutralization titers (sVNT) can be observed as early as 7 days p.i. and are sustained through day 21. Interestingly for elk calves, the mean sVNT inhibition value was 72.7% at the peak of the response, while in the adult elk the mean percent inhibition was lower at 58.4%. Virus neutralization (VN) tests were also performed for both calves and adult elk. VN titers peaked for the elk calves at 14 days p.i. with the highest titer (1:256) observed in only one calf, while in the adult elk, VN titers peaked at day 7 p.i. and did not go above 1:16 (Table 1). Altogether, these data suggest that both calves and adult elk can mount neutralizing antibody responses to SARS-CoV-2 following challenge. However, this response appears to be more robust in the calves.

**Table 1:** Virus neutralization (VN) titer results for calves and adult elk prior to and following inoculation with SARS-CoV-2.

Calves					Adults				
Animal #	PC	Day 7	Day 14	Day 21	Animal #	PC	Day 7	Day 14	Day 21
4	< 8	< 8	32	32	**2**	< 8	8	8	< 8
5	< 8	16	256	8	**3**	< 8	16	< 8	< 8
6	< 8	8	64	16	**7**	< 8	8	< 8	< 8

*PC* samples collected prior to challenge.

Blood samples collected from control animals, both calves (Supplementary table 1) and adults (Supplementary table 2), were negative for SARS-CoV-2 via sVNT.

### Viral RNA detection in nasal, oral and rectal swabs

Swabs (nasal, oral and rectal) were collected at various timepoints and analyzed via real-time RT-PCR (rRT-PCR) for the presence of viral RNA. In elk calves, nasal swabs were only found positive in 2/7 animals at day 2 p.i., in 1/5 at day 3 p.i. and in 1/3 at days 7 and 10 p.i. (Table 2). We did not observe consistent positive rRT-PCR results in swabs collected from elk calves. In contrast, we observed rRT-PCR positive results in all (7/7) inoculated adult elk at day 2 p.i., and 2/5 at days 3 and 4 p.i. (Table 3). Oral and rectal swabs did not consistently show the presence of viral RNA. No oral or rectal swab samples collected from the elk calves were positive for SARS-CoV-2 RNA, and in samples collected from the adult elk, only one oral swab was found positive in one replicate well on day 4 p.i. (Ct value 37.3) and only 2 rectal swabs were found positive on day 2 p.i. in one duplicate well for each animal (Ct values 38.1 and 36.7).

**Table 2:** Elk calves’ nasal swabs rRT-PCR results prior to and following inoculation with SARS-CoV-2.

Animal #	PC	Day 2	Day 3	Day 4	Day 5	Day 7	Day 10	Day 14	Day 21
1	ND	ND							
2	ND	ND							
3	ND	30.0/33.3	ND	ND	ND				
4	ND	ND	ND	ND	ND				
5	ND	ND	ND	ND	ND	ND	ND	ND	ND
6	ND	31.4/34.1	–/39.4	ND	ND	ND	ND	ND	ND
7	ND	ND	ND	ND	ND	36.87/–	37.9/–	ND	ND

All samples were run in duplicates. Shown are Ct values for individual duplicate wells, separated by “/”.

“–” indicates value not detected in an individual well.

*ND* is not detected in both wells, *PC* samples collected prior to challenge.

**Table 3:** Adult elk nasal swabs rRT-PCR results prior to and following inoculation with SARS-CoV-2.

Animal #	PC	Day 2	Day 3	Day 4	Day 5	Day 7	Day 10	Day 14	Day 21
1	ND	36.3/–							
2	ND	–/37.9	33.8/34.3	38.6/38.2	ND	ND	ND	ND	ND
3	ND	35.4/ND	ND	ND	ND	ND	ND	ND	ND
4	ND	29.5/31.4	ND	ND	ND				
5	ND	35.4/36.8	ND/ND	36.0/–	ND				
6	ND	34.4/36.2							
7	ND	37.2/34.3	–/38.2	ND	ND	ND	ND	ND	ND

All samples were run in duplicate. Shown are Ct values for individual duplicate wells, separated by “/” .

“–” indicates value not detected in an individual well.

*ND* not detected in both wells, *PC* samples collected prior to challenge.

Nasal, oral and rectal swabs collected from the control animals, both calves (Supplementary table 1) and adults (Supplementary table 2), were negative via rRT-PCR for SARS-CoV-2 RNA.

### Viral RNA detection in lymphoid tissues

At necropsy on days 2 and 21 p.i. medial retropharyngeal lymph node (mRPLN) and palatine tonsil samples were collected and assessed for the presence of viral RNA via rRT-PCR, based on previous white-tailed deer studies (Table 4). At day 2 p.i., SARS-CoV-2 RNA was detected in the mRPLN of one elk calf and one adult elk. In contrast, no viral RNA was detected in tonsils collected from calves or adult elk. By day 21 p.i., viral RNA was detected in all three mRPLN from both calves and adult elk. SARS-CoV-2 was also detected in 2/3 tonsil samples collected from elk calves although Ct values were high (~ 38), but none was detected in tonsils from adult elk.

**Table 4:** Viral RNA detection via rRT-PCR in tissues from elk calves and adults collected at days 2 and 21 post-infection with SARS-CoV-2.

Calves			Adult		
Necropsy	mRPLN	Tonsil	Necropsy	mRPLN	Tonsil
Day 2			D2		
#1	25.5/25.2	ND	#1	32.4/33.2	ND
#2	ND	ND	#6	ND	ND
Day 21			D21		
#5	32.2/32.6	38.7/–	#2	28.1/27.9	ND
#6	27.9/27.9	ND	#3	32.5/32.2	ND
#7	23.4/23.7	38.1/–	#7	22.5/22.9	ND

All samples were run in duplicates. Shown are Ct values for individual duplicate wells, separated by “/”.

“#” indicates animal numbers.

“–” indicates value not detected in an individual well.

*ND* is not detected in both wells, *mRLPN* medial retropharyngeal lymph node.

### Detection of SARS-CoV-2 RNA via in situ hybridization (ISH)

**Figure 2:**
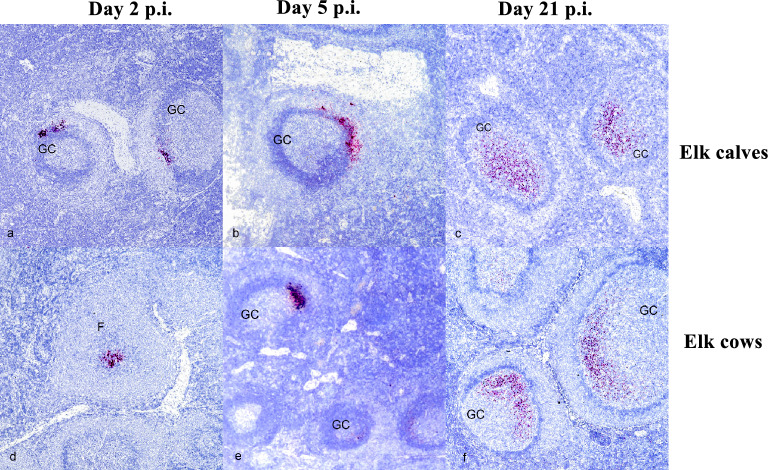
SARS-CoV-2 RNA detected via in situ hybridization (ISH) in the germinal centers of medial retropharyngeal lymph nodes of infected elk calves and cows. Medial retropharyngeal lymph nodes (mRPLN) from elk calves (**a**–**c**) and elk cows (**d**–**f**) experimentally infected with SARS-CoV-2. In both elk calves and cows, labeling was observed on 2 (**a**,**d**), 5 (**b**,**e**), and 21 (**c**,**f**) days post inoculation (p.i). Red labeling indicates presence of viral RNA within germinal centers (GC) or follicles (F). At days 2 and 5 p.i., this labeling is observed primarily within the marginal zone, while at day 21, labeling for SARS-CoV-2 RNA is seen within the GC.

Microscopic analysis of tissues collected at necropsy revealed no lesions consistent with SARS-CoV-2 infection, as reported in other species. The presence of SARS-CoV-2 RNA was investigated through ISH on various tissues. Tissues were selected based on previous studies using white-tailed deer, these included palatine tonsil, mRPLN and lung. In elk calves, viral RNA was detected in the mRPLN of 2/2 calves examined at 2 days p.i., 2/2 calves examined at 5 days p.i., and 3/3 calves examined at 21 days p.i. In adult elk, viral RNA was detected in 2/2 cows examined 2 days p.i., 2/2 cows examined 5 days p.i. and 3/3 cows examined 21 days p.i. In all cases, labeling was limited to secondary lymphoid follicles, often within germinal centers (Fig. 2). Additionally, staining for viral RNA was primarily observed within the marginal zone of the follicle in both calves and adult elk on days 2 and 5 dpi (Fig. 2a–b,d–e). However, on day 21 p.i., viral RNA staining was observed within the germinal center (Fig. 2c,f). Viral RNA was not detected in palatine tonsils or lung from inoculated calves or cows. Additionally, viral RNA was not detected in tissues examined from non-inoculated control calves and cows.

### Detection of SARS-CoV-2 protein via immunohistochemistry (IHC)

**Figure 3:**
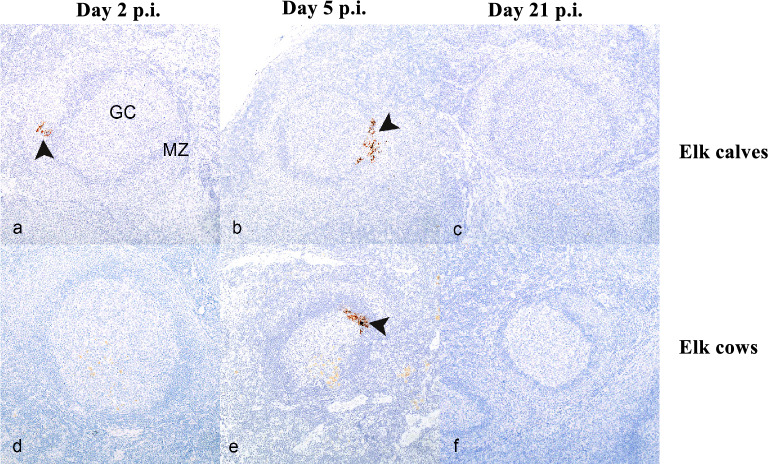
SARS-CoV-2 Spike protein detected via immunohistochemistry (IHC) in the lymphoid follicles of the medial retropharyngeal lymph nodes of infected elk. Medial retropharyngeal lymph nodes (mRPLN) from elk calves (**a**–**c**) and elk cows (**d**–**f**) experimentally infected with SARS-CoV-2 at 2 (**a**,**d**), 5 (**b**,**e**), and 21 (**c**,**f**) days post inoculation (p.i). In calves, immunolabeling was present (dark brown color at the point of arrowheads) at day 2 (**a**) and 5 p.i. (**b**), but not at day 21 p.i. (**c**). In the elk cows, no immunolabeling for SARS-CoV-2 Spike protein was observed in the lymph nodes at day 2 p.i. (**d**) or day 21 p.i. (**f**) but was present at 5 days p.i. (**e**) (arrowhead). In all cases, labeling was observed within lymphoid follicles, specifically, within the marginal zone (MZ) of follicles and not the germinal center (GC).

Given the detection of SARS-CoV-2 viral RNA via rRT-PCR and ISH, we performed IHC on mRPLN and tonsil samples in elk calves to determine if viral protein was also detectable within these tissues. In the mRPLN of elk calves, SARS-CoV-2 spike protein was detectable in 1/2 calves at 2 days p.i., 2/2 calves at 5 days p.i., and 0/3 calves at 21 days p.i (Fig. 3). No SARS-CoV-2 spike protein was detected in the tonsil of elk calves utilizing IHC. The mRPLN of adult elk were also analyzed by IHC. Immunolabeling for SARS-CoV-2 Spike protein was observed in 0/2 cows at 2 days p.i., 2/2 cows at 5 days p.i., and 0/3 cows at 21 days p.i. Staining for viral Spike protein was primarily observed within the marginal zone of the follicle.

## Discussion

Since the outbreak of the 2019 SARS-CoV-2 pandemic, concerns regarding the susceptibility of other vertebrate species, and their potential to act as reservoirs for the virus, have garnered attention. Field surveillance as well as various experimental infection studies have demonstrated a wide range of susceptibility across various species in the families Felidae, Canidae, Mustelidae, Cricetidae, and Cervidae^22,23^. Among these, white-tailed deer have emerged as a species of interest given their susceptibility to infection, the high seroprevalence in free-ranging populations, their ability to transmit the virus to other deer as well as back to humans, and the identification of novel variants within this species^1–3,7–10,13^.

Characterization of susceptible wildlife species is critical for understanding not only the epidemiology of this virus, but also for our ability to implement intervention strategies to stop the spread of this disease. In this work, we sought to characterize the susceptibility of North American elk to SARS-CoV-2 infection, another cervid species with broad distribution in the US. Here, we demonstrate that both elk calves and adult elk are susceptible to infection with the ancestral Wuhan-like variant of SARS-CoV-2 (USA-WA1/2020), as characterized by the development of measurable neutralizing antibody responses and the detection of viral RNA and viral protein in the retropharyngeal lymph nodes of infected animals. However, this work demonstrates that there may be some differences in the quality of the responses between the two age groups.

A previous study by Porter et al.^21^ demonstrated that weanling North American elk are minimally susceptible to infection with the Delta variant of SARS-CoV-2. Post challenge, viral RNA could be detected from oral and nasal swabs between days 1 and 5 p.i. However, infected elk calves did not shed infectious virus, nor were they capable of transmission to an in-contact elk. Neutralizing antibody responses were observed in these animals, however, these responses were relatively low, with peak neutralizing titers at 1:20 at 21 days p.i. Additionally, no infectious virus was detected in tissues collected at necropsy. Similar to these findings, we observed the presence of viral RNA from nasal swabs and the development of neutralizing titers from infected animals. However, and in contrast to the results observed with Delta variant infection, we observed peak neutralizing titers at 14 days p.i., with values ranging from 1:32 up to 1:256 (Table 1). Interestingly, when we compared elk calves to adult virus neutralization titers, the elk calves appear to have a higher neutralizing response as adult elk responses remained at 1:8 or 1:< 8 at all timepoints except for one animal at day 7 p.i. with a 1:16 titer.

Differences in viral neutralization responses may be related to the age of the animals and exposure to other viruses, including other coronaviruses. The high homology between SARS-CoV-2 and other coronaviruses may result in cross-reactive immune responses. In humans, cross-reactive humoral and cellular immune responses developed prior to SARS-CoV-2 infection, have been characterized and demonstrated to play an important role in determining susceptibility to infection and disease progression (reviewed in^24^). Pre-existing cross-reactive responses from previous viral infections may be beneficial or detrimental. For example, cross-reactive antibodies to one virus may bind a to another virus and provide neutralization and subsequent clearance. Alternatively, pre-existing cross-reactive antibodies to a virus may bind to another virus with low-avidity resulting in enhanced viral uptake via antibody-mediated internalization and/or inhibit the generation of de novo antibody responses to the latter, leading to increased viral load^24^. Our data suggests that adult elk appear to have a lower magnitude of neutralizing antibody responses (both in sVNT and VN) as compared to the elk calves. These slight differences may be attributed to some level of pre-existing immunity in older animals, which allows them to control the infection more rapidly, thus decreasing viral load available for de novo antibody responses. Interestingly, in the adult elk we find that sVNT inhibition values increase over the time course, and yet VN values do not follow the same trend. Discrepancies between sVNT and VN values can be explained by the presence of antibodies that able to interfere with the interaction between the Spike protein receptor binding domain (RBD) and the ACE2 receptor, but are unable to block virus entry^25^. These data would suggest that while adult elk produce de novo antibodies to the Spike protein, they may not have a neutralizing function. However, this does not mean that they cannot control the infection. Heterogeneity in humoral responses in humans, including higher ratios of anti-Spike to anti-Nucleocapsid antibody and antibodies with different functional capacity (i.e. phagocytic, complement activating), have been characterized in patients with favorable outcomes^26^. We can speculate that functionally, the antibody response detected via sVNT in adult elk may not be neutralizing but could have alternative functions in controlling disease.

Persistence of viral RNA, detectable by rRT-PCR and ISH, in tissues such as the mRPLNs is consistent with previous studies in white-tailed deer, including the location of labeling within secondary lymphoid follicles and germinal centers of lymphoid tissues^1,27^. Similarly, persistent SARS-CoV-2 RNA has been detected in human lymph nodes, tonsils and other tissues at autopsies conducted over 300 days post infection^28^. In such cases, both in deer species and humans, it was not possible to isolate virus from these tissues, although viral RNA was detected using ISH or rRT-PCR. A potential explanation for the detection of viral RNA in the absence of infectious virus is the integration of viral subgenomic RNA into the DNA of the host cell via reverse transcription. Ancestral evidence of non-retroviral RNA virus sequences in the genome of vertebrate species have been previously detected^29,30^. Additionally, DNA copies of nonretroviral RNA viruses including lymphocytic choriomeningitis virus (LCMV) and vesicular stomatitis virus have been shown to integrate into the DNA of their host cell^31–33^. Recently, using cultured human cells Zhang et al.^34^ demonstrated that SARS-CoV-2 can integrate into the genome of host cells. Additionally, using published RNAseq data from cultured cells and organoid tissues, they demonstrate that SARS-CoV-2 sequences integrated into the host cell genome can be expressed as human-viral chimeric reads. RNA expression of viral subgenomic sequences could explain why some patients remain rRT-PCR positive for SARS-CoV-2 many weeks or months following recovery from infection. While infectious virus cannot be produced from this integrated material, it does raise the possibility that viral antigen could be expressed by host cells. In the work presented here we demonstrate the presence of viral RNA 21 days p.i. with peak viral Spike protein production at day 5 p.i. As detection of Spike protein wanes, viral RNA is consistently detected via ISH and rRT-PCR. The lack of correlation between viral RNA and protein production would suggest that active translation is not occurring. Additionally, based on nasal and oral swab data, we did not detect viral RNA in these secretions. These data would suggest that viral replication and shedding is not occurring. Consistent with continuous or recurrent SARS-CoV-2 rRT-PCR positive reports in recovered human patients^35,36^, the persistent viral RNA found in the lymph nodes of other susceptible species including white-tailed deer^1,2^ and elk, as demonstrated here, may point to a common mechanism of viral persistence in susceptible hosts. However, the immunological implications of this phenomenon are not completely understood and warrant further study.

The work presented here focuses on the susceptibility of elk to infection, with the ancestral Wuhan-like variant of SARS-CoV-2. With no field information regarding any circulating strains of SARS-CoV-2 in free-ranging elk, we opted to use a strain that would be considered less human-adapted as compared to Delta or Omicron. The data obtained from these studies demonstrate that both calves and adult North American elk are susceptible to infection with this ancestral variant of SARS-CoV-2, as they permit viral replication and develop virus neutralizing antibody responses following infection. However, no clinical signs of disease nor pathological changes in the lungs or lymph nodes of infected animals were observed, suggesting that they are not susceptible to disease. Interestingly, our findings suggest that there is persistence of SARS-CoV-2 viral RNA in the lymphoid tissues of infected animals in the presence of an immune response. The continued assessment of SARS-CoV-2 susceptibility in various species provides insights not only into potential reservoirs for the disease but also, as shown here, sheds light on the host–pathogen interactions that may be common across species and may drive immunity to infection and disease.

## Materials and methods

### Cells and virus

Vero E6 (ATCC® CRL-1586™) cells were cultured in Eagle’s Minimum Essential Medium (EMEM, ATCC) supplemented with 10% fetal bovine serum (FBS) and 1% antibiotic–antimycotic 100X (Gibco™, Life Technologies, Carlsbad, CA, USA). The cell cultures were maintained at 37 °C with 5% CO_2_. The SARS-CoV-2 isolate (USA-WA1/2020) was obtained from BEI Resources (SARS-Related Coronavirus 2, Isolate hCoV-19/USA-WA1/2020, NR-52281, Lot#70036318). The stock virus was passaged 3 times in Vero E6 cells, clarified by centrifugation (1000 rpm for 5 min) and stored at − 80 °C. Viral titer was determined by the Reed and Muench^37^. A viral suspension containing 10^5.5^ tissue culture infectious dose 50 per ml (TCID_50_/ml) was used for elk calf inoculations and 10^6^ TCID_50_/ml for adult elk inoculations.

### Animal infection and sampling

All animal work and procedures were approved prior to the experiment by the National Animal Disease Center (NADC) Institutional Animal and Care Use Committee (IACUC) (protocol #ARS-22-1047). Additionally, all methods were performed according to the IACUC and the Guide for Care and Use of Laboratory Animals guidelines and regulations. Elk calves (~ 5 months old; n = 11) and adult elk cows (~ 4 years old; n = 10) were obtained from a captive herd at the NADC in Ames, IA. Calves used in the challenge study were weaned at approximately 3 months of age and housed separately from the adults post-weaning. Control calves remained with the herd. For the study, animals were housed in an agriculture biosafety level 3 (ABSL-3) facility at NADC and allowed to acclimate for a minimum of 2 weeks. Calf and adult experiments were performed independent of one another.

Animals to be inoculated were sampled and screened for SARS-CoV-2 RNA by rRT-PCR in oronasal secretions and by surrogate virus neutralization test (sVNT) and VN assays prior to virus inoculation. Seven elk calves and 7 cows were sedated with a combination of xylazine and ketamine and intranasally inoculated with an atomization device (LMA® MAD Nasal™, Teleflex; Morrisville, NC, USA) for delivery of approximately 2.5 mL of inoculum into each nostril for a total of 5 mL. Following inoculation, the effects of xylazine were reversed using tolazoline. On days 0, 2, 3, 4, 5, 7, 10, 14 and 21 post-inoculation (p.i.) elk were sedated as described above and nasal, oral and rectal swabs collected for rRT-PCR. Blood was collected on days 0, 7, 14 and 21 days p.i. for serologic assays. On days 2 and 5 p.i. 2 calves and 2 cows each were euthanized and examined. All other inoculated elk were euthanized and examined 21 days p.i.

Four calves and 3 cows remained as non-inoculated controls, housed separately from challenged animals. All controls animals were sampled and screened for SARS-CoV-2 RNA by rRT-PCR in nasal, oral, and rectal swabs and by surrogate virus neutralization test (sVNT). Animals were euthanized and examined at necropsy similar to inoculated elk.

### Serology

The cPASS SARS-CoV-2 neutralization antibody detection kit (GenScript Biotech, Amsterdam, Netherlands) was used as described^38,39^ and according to the manufacturer’s recommendations. The assay detects the presence of specific anti-SARS-CoV-2 neutralizing antibodies against the Spike protein in serum in a species and isotype-independent manner by blocking the interaction between the receptor-binding domains (RBD) of the viral Spike glycoprotein with the ACE2 cell surface receptor. Spectrophotometry was conducted at 450 nm in a plate reader. The absorbance of the sample is inversely dependent on the titer of the anti-SARS-CoV-2 neutralizing antibodies in tested samples. To confirm the sVNT results, serum samples were submitted to the National Veterinary Services Laboratory (NVSL, Ames, Iowa) for testing via virus neutralization (VN). Briefly, serum was serially diluted twofold with a starting dilution of 1:8. Each dilution was incubated with virus for one hour at 37 °C. The virus had a TCID_50_ of 100. Vero-76 cell culture was then added to the virus/serum mixture and incubated at 37 °C for 3 days. Each well was observed for presence of absence of cytopathic effect.

### Real-time RT-PCR (rRT-PCR) on swabs and tissues

To assess for viral shedding on nasal, oral and rectal samples, swabs were submitted to NVSL for processing and for rRT-PCR analysis.

To determine the presence of viral RNA in tissue samples, tissues were thawed, cut into an approximately 50–100 mg piece, and resuspended in 1 -2 mL of TRI-Reagent® (Life Technologies, Carlsbad, CA, USA) in individual gentle MACS™ M tubes (Miltenyi Biotec, Bergisch Gladbach, Germany). Tissues were dissociated using a gentle MACS™ Octo-Dissociator (Miltenyi Biotec) following the manufacturer’s recommendations. RNA was extracted from tissue homogenate samples using the MagMAX™-96 for Microarrays Total RNA Isolation Kit (Applied Biosystems, Waltham, MA, USA). Samples were run on a MagMAX™ Express Magnetic Particle Processor (Applied Biosystems) following the manufacturer’s instructions. Next, 15 µL of extracted product was added to 5 µL of the AgPath-ID™ One step RT-PCR master mix (Applied Biosystems). Samples were run in duplicate. The RT-qPCR reactions were performed on an ABI 7500 Fast instrument (Applied Biosystems) run in standard mode with the following conditions: 1 cycle at 45 °C for 10 min, followed by 1 cycle at 95 °C for 10 min, 1 cycle at 95 °C for 3 s, and 45 cycles at 55 °C for 30 s. The forward primer sequence was 5′-GACCCCAAAATCAGCGAAAT-3′, the reverse primer sequence was 5′-TCTGGTTACTGCCAGTTGAATCTG-3′, and the probe sequence was 5′-FAM-ACCCCGCATTACGTTTGGTGGACC-BHQ1-3′, targeting the nucleocapsid (N) gene. A positive control (2019-nCoV_N_Positive Control, Integrated DNA Technologies IDT, Coralville, IA, USA) and a negative control was run on every plate.

For all rRT-PCR assays, samples with a cycle threshold (Ct) of ≤ 40 were considered positive.

### Necropsy and sample collection

Two inoculated calves and 2 inoculated adult cows were euthanized on days 2 and 5 p.i. and the remaining animals were euthanized on day 21 p.i. Following necropsy, multiple tissues (palatine tonsil, nasal turbinate, medial retropharyngeal lymph node, cerebellum, cerebrum, olfactory lobes, caudate nucleus, trachea, lung [right and left cranial and caudal lobes], heart, tracheobronchial lymph node, mediastinal lymph node, liver, spleen, kidney) were collected. Samples were individually bagged, placed on dry ice, and transferred to a − 80 °C freezer until testing. Additionally, tissue samples were collected and processed for standard microscopic examination, a subset were also processed by in situ hybridization (ISH) and immunohistochemistry (IHC). For this, tissue sections of approximately ≤ 0.5 cm in width were fixed by immersion in 10% neutral buffered formalin (≥ 20 volumes fixative to 1 volume tissue) for approximately 24 h, and then transferred to 70% ethanol, followed by standard paraffin embedding techniques. Slides for standard microscopic examination were stained with hematoxylin and eosin (HE).

### In situ hybridization (ISH)

Paraffin-embedded tissues were sectioned at 5 µm and subjected to ISH using the RNAscope ZZ probe technology (Advanced Cell Diagnostics, Newark, CA). In situ hybridization was performed to detect tissue distribution of SARS-CoV-2 RNA in tissues. Palatine tonsil, mRPLN, and lung were tested by RNAscope 2.5 HD Reagents–RED kit (Advanced Cell Diagnostics) as previously described^27^. Proprietary ZZ probes targeting SARS-CoV-2 RNA (V-nCoV2019-S probe) designed and manufactured by Advance Cell Diagnostics were used for detection of viral RNA. As positive controls, a probe targeted to the *Bos taurus*–specific cyclophilin B (PPIB) gene and a non-species-specific probe targeting the ubiquitin (UBC) gene were used. As a negative control, a probe targeting dapB of *Bacillus subtilis* was used. 

### Immunohistochemistry

Immunohistochemical staining for SARS-CoV-2 was performed on palatine tonsils from calves and mRPLN from calves and adult elk. To prepare the formalin-fixed paraffin-embedded tissues for staining they were heated for 45 min at 57 °C. Tissues were then deparaffinized with xylene and rehydrated through a series of graded alcohol solutions. Slides were submerged in a 1X citrate unmasking solution (Abcam) until boiling was initiated, then maintained in the unmasking solution at a sub-boiling temperature (95–98 °C) for ten minutes to perform epitope retrieval. A 3% hydrogen peroxide solution (Fischer Bioreagents, catalog no. BP2633500) was used to quench endogenous peroxidases. Slides were then immersed in a blocking solution of Tris Buffered Saline (Thermo Scientific) and Tween20® (Sigma-Aldrich) with 5% normalized goat serum. A rabbit monoclonal antibody targeting the Spike protein of SARS-CoV-2 (S1) at a concentration of 1:800 was used as the primary antibody (Cell Signaling Technologies, Boston, MA). Tissues were then incubated in a SignalStain® Boost IHC Detection Reagent (HRP, Mouse, Cell Signaling, Catalog No. 8125S) followed by SignalStain® DAB substrate to produce a brown reaction product (Cell Signaling Technologies). Finally, counterstaining was performed using hematoxylin stain solution and Bluing Agent (Ventana). Nasal turbinate tissue from a single mink inoculated with SARS-CoV-2 served as a positive control.

### Ethics statement

All animal work presented in this manuscript was performed under approval of the National Animal Disease Center (NADC) Institutional Animal Care and Use Committee (IACUC). The study is reported in accordance with ARRIVE guidelines.

### Supplementary Information

Supplementary Tables.

## Data Availability

Data is provided within the manuscript.

## References

[1] Martins M (2022). From Deer-to-Deer: SARS-CoV-2 is efficiently transmitted and presents broad tissue tropism and replication sites in white-tailed deer. PLoS Pathog..

[2] Palmer MV (2021). Susceptibility of white-tailed deer (*Odocoileus virginianus*) to SARS-CoV-2. J. Virol..

[3] Chandler JC (2021). SARS-CoV-2 exposure in wild white-tailed deer (*Odocoileus virginianus*). Proc. Natl. Acad. Sci. USA.

[4] Hale VL (2022). SARS-CoV-2 infection in free-ranging white-tailed deer. Nature.

[5] Kuchipudi SV (2022). Multiple spillovers from humans and onward transmission of SARS-CoV-2 in white-tailed deer. Proc. Natl. Acad. Sci. USA.

[6] Palermo PM, Orbegozo J, Watts DM, Morrill JC (2022). SARS-CoV-2 neutralizing antibodies in white-tailed deer from Texas. Vector Borne Zoonotic Dis..

[7] Roundy CM (2022). High seroprevalence of SARS-CoV-2 in white-tailed deer (*Odocoileus virginianus*) at one of three captive cervid facilities in Texas. Microbiol. Spectr..

[8] Vandegrift KJ (2022). Detection of SARS-CoV-2 Omicron variant (B.1.1.529) infection of white-tailed deer. bioRxiv.

[9] Pickering B (2022). Divergent SARS-CoV-2 variant emerges in white-tailed deer with deer-to-human transmission. Nat. Microbiol..

[10] Willgert K (2022). Transmission history of SARS-CoV-2 in humans and white-tailed deer. Sci. Rep..

[11] Damas J (2020). Broad host range of SARS-CoV-2 predicted by comparative and structural analysis of ACE2 in vertebrates. Proc. Natl. Acad. Sci. USA.

[12] Lopes LR (2023). Cervids ACE2 residues that bind the spike protein can provide susceptibility to SARS-CoV-2. Ecohealth.

[13] Holding M (2022). Screening of wild deer populations for exposure to SARS-CoV-2 in the United Kingdom, 2020–2021. Transbound. Emerg. Dis..

[14] Krupinska M (2022). Wild Red Deer (*Cervus elaphus*) do not play a role as vectors or reservoirs of SARS-CoV-2 in North-Eastern Poland. Viruses.

[15] Moreira-Soto A (2022). Serological evidence that SARS-CoV-2 has not emerged in deer in Germany or Austria during the COVID-19 pandemic. Microorganisms.

[16] Foundation, R. M. E. *Elk Fact sheet*, <rmef.org/elk-facts/> (2024).

[17] Cotterill GG (2018). Winter feeding of elk in the Greater Yellowstone Ecosystem and its effects on disease dynamics. Philos. Trans. R. Soc. Lond. B Biol. Sci..

[18] Kohl M.T., C. S. M., Ellis C.C., Halseth A.N., Merkle J.A., Proffitt K.M., Rowland M.M., Wisdom M.J. in *Rangeland Wildlife Ecology and Conservation* (ed Dahlgren D.K. McNew L.B., Beck J.L.) Ch. 20, 703–733 (Springer Nature Switzerland, 2023).

[19] Franklin AB (2013). Wild ungulates as disseminators of Shiga toxin-producing *Escherichia coli* in urban areas. PLoS ONE.

[20] Olsen SJ (2002). A waterborne outbreak of *Escherichia coli* O157:H7 infections and hemolytic uremic syndrome: Implications for rural water systems. Emerg. Infect. Dis..

[21] Porter SM (2024). Experimental SARS-CoV-2 infection of elk and mule deer. Emerg. Infect. Dis..

[22] Frazzini S, Amadori M, Turin L, Riva F (2022). SARS CoV-2 infections in animals, two years into the pandemic. Arch. Virol..

[23] Hobbs EC, Reid TJ (2021). Animals and SARS-CoV-2: Species susceptibility and viral transmission in experimental and natural conditions, and the potential implications for community transmission. Transbound. Emerg. Dis..

[24] Murray SM (2023). The impact of pre-existing cross-reactive immunity on SARS-CoV-2 infection and vaccine responses. Nat. Rev. Immunol..

[25] Saker K (2022). Evaluation of commercial anti-SARS-CoV-2 neutralizing antibody assays in seropositive subjects. J. Clin. Virol..

[26] Roltgen K (2020). Defining the features and duration of antibody responses to SARS-CoV-2 infection associated with disease severity and outcome. Sci. Immunol..

[27] Palmer MV (2021). Susceptibility of white-tailed deer (*Odocoileus virginianus*) to SARS-CoV-2. J. Virol..

[28] Proal AD (2023). SARS-CoV-2 reservoir in post-acute sequelae of COVID-19 (PASC). Nat. Immunol..

[29] Belyi VA, Levine AJ, Skalka AM (2010). Unexpected inheritance: multiple integrations of ancient bornavirus and ebolavirus/marburgvirus sequences in vertebrate genomes. PLoS Pathog.

[30] Horie M (2010). Endogenous non-retroviral RNA virus elements in mammalian genomes. Nature.

[31] Geuking MB (2009). Recombination of retrotransposon and exogenous RNA virus results in nonretroviral cDNA integration. Science.

[32] Klenerman P, Hengartner H, Zinkernagel RM (1997). A non-retroviral RNA virus persists in DNA form. Nature.

[33] Shimizu A (2014). Characterisation of cytoplasmic DNA complementary to non-retroviral RNA viruses in human cells. Sci. Rep..

[34] Zhang L (2021). Reverse-transcribed SARS-CoV-2 RNA can integrate into the genome of cultured human cells and can be expressed in patient-derived tissues. Proc. Natl. Acad. Sci. USA.

[35] Li N, Wang X, Lv T (2020). Prolonged SARS-CoV-2 RNA shedding: Not a rare phenomenon. J. Med. Virol..

[36] Yang JR (2020). Persistent viral RNA positivity during the recovery period of a patient with SARS-CoV-2 infection. J. Med. Virol..

[37] Reed LJ, Muench HA (1938). A simple method of estimating fifty percent endpoints. Am. J. Hyg..

[38] Tan CW (2020). A SARS-CoV-2 surrogate virus neutralization test based on antibody-mediated blockage of ACE2-spike protein-protein interaction. Nat. Biotechnol..

[39] Jemersic L (2021). Investigating the presence of SARS CoV-2 in free-living and captive animals. Pathogens.

