# Supplementary material for: Persistence of viral RNA in North American elk experimentally infected with an ancestral strain of severe acute respiratory syndrome coronavirus 2 (SARS-CoV-2)
Source: Sci Rep. 2024 May 15;14:11171. doi: 10.1038/s41598-024-61414-7 (PMC11096316; doi:10.1038/s41598-024-61414-7)
Supplement: Supplementary file 1 — Supplementary Tables. [file 41598_2024_61414_MOESM1_ESM.pdf]

Persistence of viral RNA in North American elk experimentally infected with an ancestral strain of severe acute respiratory syndrome coronavirus 2 (SARS-CoV-2)

Paola M. Boggiatto, Alexandra Buckley, Eric D. Cassmann, Hannah Seger, Steven C. Olsen, Mitchell V. Palmer

Supplementary table 1. Serology (sVNT) and rRT-PCR results from control elk calves.

Animal #	Calves			
	sVNT	rRT-PCR nasal swab	rRT-PCR oral swab	rRT-PCR rectal swab
8	-4.3	ND	ND	ND
9	8.2	ND	ND	ND
10	10.5	ND	ND	ND
11	8.06	ND	ND	ND

All rRT-PCR samples were run in duplicates.
"--" indicates value not detected in an individual well.
"ND" is not detected in both wells.

Supplementary table 2. Serology (sVNT) and rRT-PCR results from control adult elk prior.

Animal #	Adults			
	sVNT	rRT-PCR nasal swab	rRT-PCR oral swab	rRT-PCR rectal swab
8	6.17	ND	ND	ND
9	18.1	ND	ND	ND
10	14.9	ND	ND	ND

All rRT-PCR samples were run in duplicates.
"--" indicates value not detected in an individual well.
"ND" is not detected in both wells.
